# Congenital unilateral pericardial agenesis presenting as an isolated chest pain in an adolescent: a case report and comprehensive review

**DOI:** 10.1186/s13019-025-03364-3

**Published:** 2025-02-15

**Authors:** Farshad Jafari, Maryam Taheri, Pouya Ebrahimi, Maedeh Soflaee, Reyhaneh Alipore Rafie, Mohsen Anafje

**Affiliations:** 1 Pediatric Interventional Cardiologist, Rajaie Cardiovascular Medical and Research Institute, Iran University of Medical Sciences, Tehran, Iran; 2 Cardiovascular Diseases Research Institute, Tehran Heart Center, Tehran University of Medical Sciences, Tehran, Iran; 3 Rajaie Cardiovascular Medical and Research Institute, School of Medicine, Iran University of Medical Sciences, Tehran, 1995614331 Iran

**Keywords:** Congenital pericardial agenesis, Chest pain, Pericardium, Absent, Case report

## Abstract

**Introduction:**

Congenital pericardial agenesis is a rare congenital anomaly resulting from the incomplete development of the pleuropericardial membranes during embryogenesis, leading to the partial or complete absence of the pericardial sac. Although this condition usually remains asymptomatic, it can present with various misleading symptoms such as chest pain (add some other, maybe 2, more prevalent presenting symptoms), making diagnosis challenging. Advanced imaging techniques are crucial for accurate diagnosis and management, especially when usual diagnostic modalities do not achieve a definite diagnosis.

**Case presentation:**

The history and diagnostic process of a 16-year-old female who presented with isolated, non-exertional chest pain are detailed. A comprehensive diagnostic work-up was initiated, including chest X-ray (CXR), transthoracic echocardiogram (TTE), CT angiography (CTA), and cardiac magnetic resonance imaging (CMRI). These advanced imaging modalities unveiled the rare and elusive diagnosis of left-sided pericardial agenesis, decisively ruling out other potential causes and shedding light on an extraordinary case that challenges conventional diagnostic pathways.

**Conclusion:**

Unilateral pericardial agenesis, though typically benign and often shrouded in clinical obscurity, can manifest with enigmatic symptoms such as isolated chest pain, necessitating a meticulous and comprehensive diagnostic approach. Multimodal imaging is essential for accurate diagnosis and for ruling out complications such as cardiac herniation or coronary artery compression. Considering the absence of significant complications, conservative management was chosen in this case, with the patient being discharged with instructions to monitor for any warning signs.

**Clinical key message:**

Clinicians should consider congenital pericardial agenesis as one of the potential causes of unexplained chest pain, particularly when the initial investigations are inconclusive. Advanced imaging techniques (such as CXR and MRI) are vital for confirming the diagnosis and subsequently appropriate and timely management and preventing potential complications.

## Introduction

Congenital agenesis of the pericardium, a rare and frequently asymptomatic anomaly, arises from defective development of the pleuropericardial membranes during embryogenesis, leading to partial or complete absence of the pericardial sac [1]. When symptomatic, it can present with complications such as cardiac chamber herniation or coronary artery compression, underscoring the critical role of advanced imaging techniques in accurate diagnosis and management​ [2]. Congenital pericardial agenesis, first described in 1559 by anatomist Realdo Colombo, is an exceedingly rare condition with an estimated incidence of less than 1 in 10,000, and its true prevalence may be underestimated due to the frequent asymptomatic nature and diagnostic challenges associated with the disorder​ [3]. The condition can manifest in several forms, including the complete absence of the pericardium, which is the most common, as well as partial absence on the left or right side, where the size of the defect can range from a small foramen to an extensive defect [4]. Moreover, Congenital absence of the pericardium is often associated with other congenital anomalies, such as atrial septal defects, patent ductus arteriosus, mitral valve disease, and tetralogy of Fallot, with 30–50% of patients exhibiting these or other anomalies, highlighting the importance of comprehensive evaluation in affected patients [5]. Non-cardiac anomalies associated with congenital pericardial agenesis include pectus excavatum, diaphragmatic hernia, and associations with syndromes such as VACTERL and Pallister–Killian, underscoring the diverse range of potential abnormalities that can coexist with this rare condition​ [6]. In this report, we present the case of a 16-year-old female who presented with isolated chest pain, leading to the diagnosis of complete left-sided pericardial agenesis.

## Case presentation

A 16-year-old female presented to the urgent care clinic with a complaint of sharp, intermittent, non-exertional chest pain localized to the left anterior chest. The pain had a sudden onset at rest and persisted for several hours before presentation. The patient denied any radiation of the pain, and there were no associated symptoms such as shortness of breath, palpitations, syncope, or dizziness. The pain was not relieved by over-the-counter analgesics and was unaffected by changes in position or respiration. She reported no history of fever, cough, recent infections, or any other symptoms suggestive of systemic or respiratory conditions. There was no history of previous trauma or recent physical exertion, and she had not experienced similar episodes in the past. Her medical history was unremarkable, with no significant illnesses or chronic conditions. She was not on any medications, had no known drug allergies, and her family history was negative for cardiac or congenital anomalies, sudden cardiac death, or other heritable conditions. Socially, the patient was a non-smoker, denied alcohol or illicit drug use, and reported no recent stress or psychological distress.

On physical examination, the patient appeared alert and in no acute distress. Her vital signs were as follows: heart rate 72 beats per minute, blood pressure 118/72 mmHg, respiratory rate 16 breaths per minute, oxygen saturation 98% on room air, and she was afebrile. The cardiovascular examination was normal, with a regular heart rhythm, normal heart sounds, and no murmurs, rubs, or gallops. There was no evidence of jugular venous distension, peripheral edema, or cyanosis, and peripheral pulses were symmetrical and palpable. The respiratory examination showed clear breath sounds bilaterally, with no wheezing, crackles, or rhonchi, and normal chest expansion. The gastrointestinal examination was unremarkable, with a soft, non-tender abdomen, no organomegaly, and normal bowel sounds. Musculoskeletal examination revealed no chest wall tenderness, and the thoracic cage appeared normal. The patient had a full range of motion in the upper limbs, with no evidence of joint or muscle abnormalities. Neurological examination was unremarkable, with intact cranial nerves and normal motor and sensory functions.

## Method

**Fig. 1:**
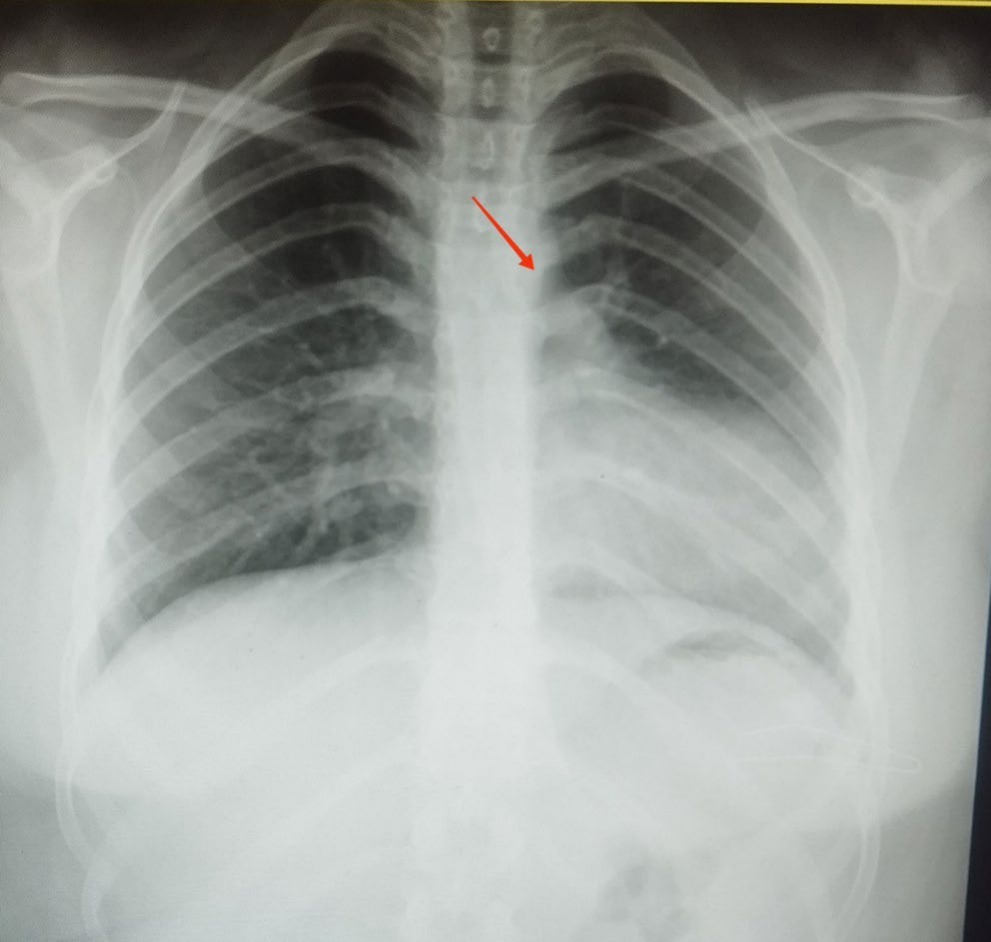
Mediastinal shift to the left side with loss of the right heart border silhouette, hyperinflation of the right lung, and reduced left lung volume. Note the interposition of lung tissue between the aorta and pulmonary artery (red arrow)

**Fig. 2:**
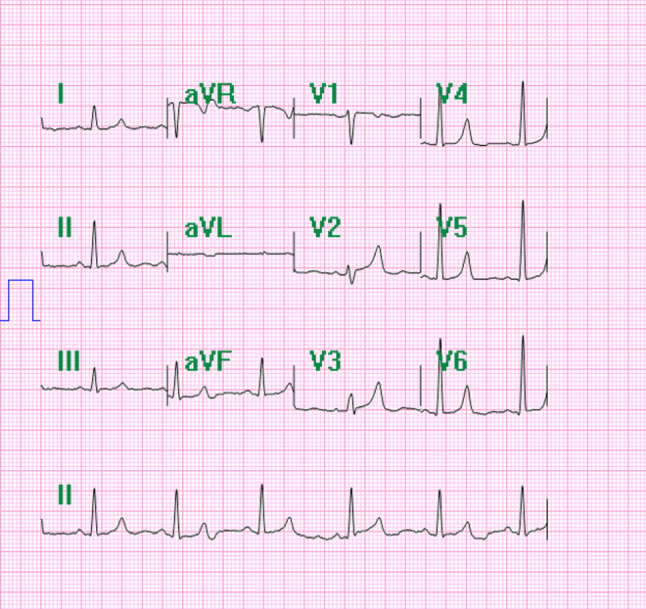
The electrocardiogram showed unspecific ST-T segment change in leads V2 and V3

Upon initial presentation, a comprehensive evaluation was initiated to determine the cause of the patient’s chest pain. The first step involved a chest X-ray (CXR), which revealed an abnormal cardiac silhouette with a noticeable leftward displacement of the heart, suggestive of a possible pericardial abnormality (Fig. 1). To further investigate this finding, a series of laboratory tests were performed, including a complete blood count, basic metabolic panel, and inflammatory markers, all returned within normal limits, effectively ruling out infectious or inflammatory causes of chest pain. The electrocardiogram showed nonspecific changes in leads V2 and V3 (Fig. 2).

**Fig. 3:**
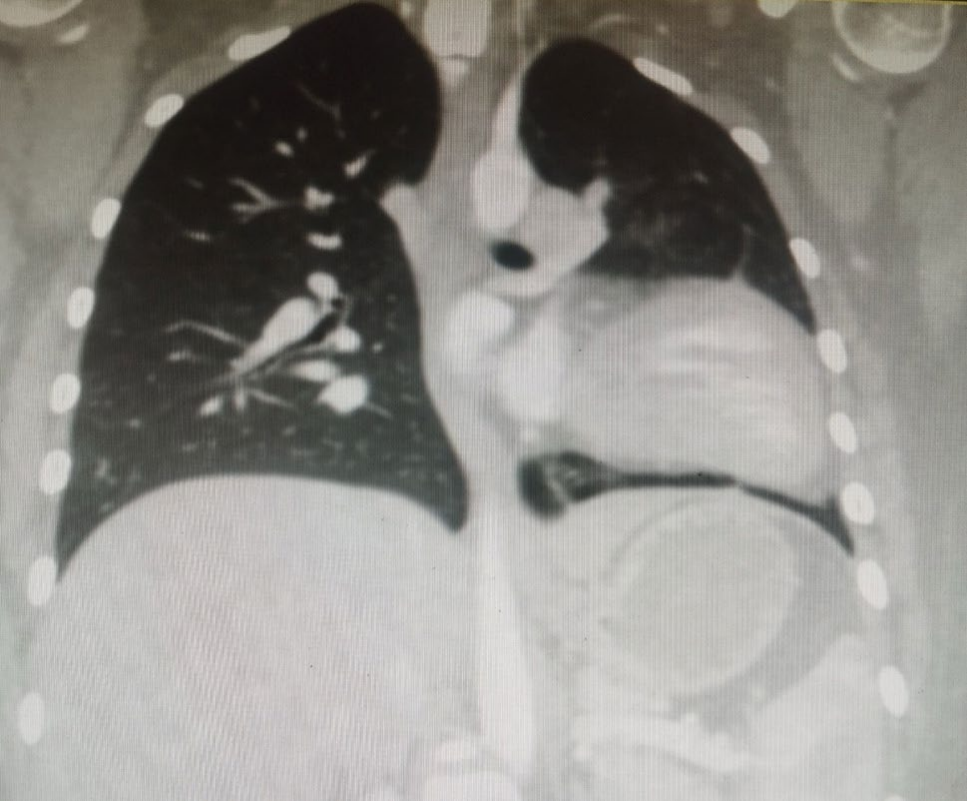
Chest CT Scan (Coronal View): Lung parenchyma is visible between the left hemidiaphragm and the base of the heart. Hyperinflation of the right lung and reduced volume of the left lung are noted

**Fig. 4:**
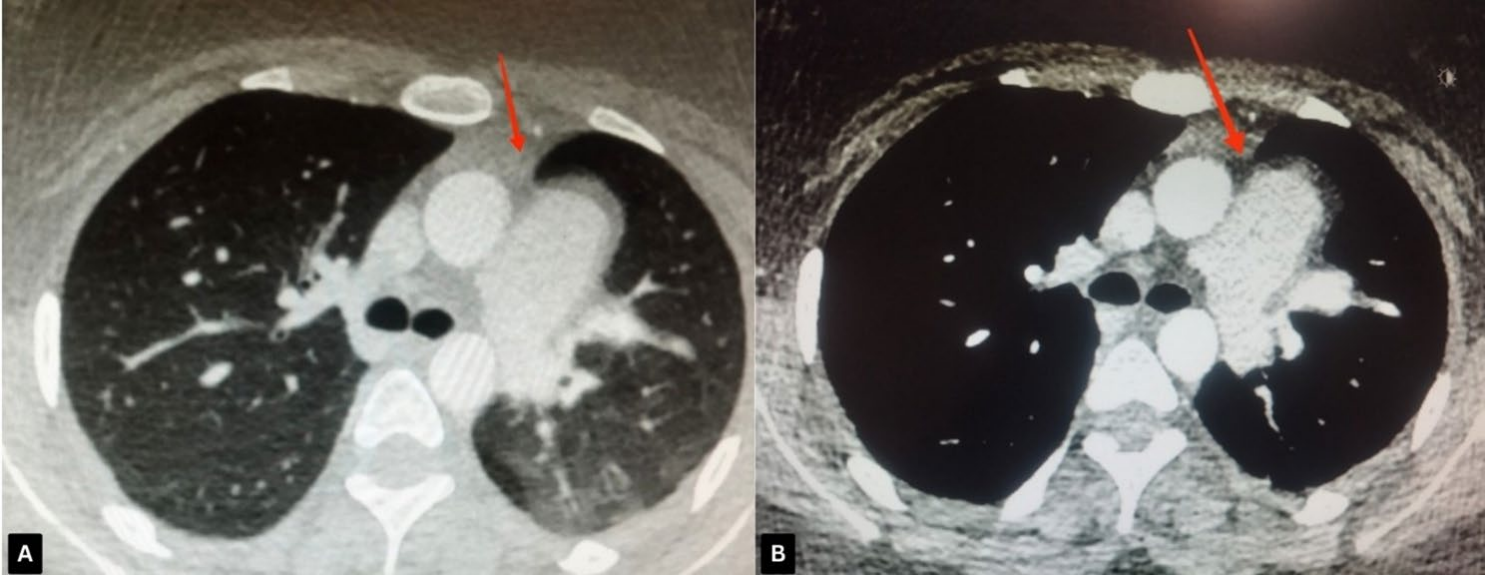
Chest CT Scan (Axial View): Lung tissue is detected between the aorta and the main segment of the pulmonary artery (red arrow). Mosaic attenuation is observed in the upper lobe of the left lung (**A** and **B**)

Given the chest X-ray findings and persistent symptoms, a transthoracic echocardiogram (TTE) was conducted. The echocardiogram demonstrated normal left and right ventricular function but revealed an unusual cardiac position within the thoracic cavity, raising suspicion of a structural anomaly. Notably, there was no evidence of pericardial effusion or other significant abnormalities on the echocardiogram (Table 1). To better assess the heart’s anatomy and rule out coronary artery disease, a CT angiography was conducted. The scan confirmed the abnormal position of the heart and revealed an absence of the pericardium on the left side (Fig. 3). Additionally, the scan showed lung tissue interposed between the aorta and pulmonary artery, hyperinflation of the right lung, and reduced volume of the left lung. There was no evidence of coronary artery disease or other vascular anomalies (Fig. 4) and (Table 1).

**Table 1:** Comprehensive diagnostic Imaging findings in the patient

Examination	Findings
**TTE**	
Systemic Veins	Normal size SVC & IVC, Increased turbulence at SVC ostium (PPg = 9mmHg, MPG = 5mmHg), Suspicious of external compression
Pulmonary Veins	3PVs seen with normal return to LA
Cardiac Chambers	Mild to moderate RAE, RVE, LAE, LVE, Hypokinetic septum, LVEF = 50%, No RVH, No LVH
Atypical Cardiac Position	Leftward displacement and elongation of the heart within the thoracic cavity, with associated interposition of lung tissue between the heart and diaphragm
Atrioventricular Valves	Mild MR, TR (G = 23mmHg)
Great Arteries & Valves	NRGAS, TAV, AO ann = 2.1 cm, AO VTI = 21.7 cm, PA ann = 2.65 cm, Qp/Qs = 1.3, mild PI (PPG = 14mmHg), RPA = 1.6 cm, LPA = 1.4 cm, Normal coronary origin
Septum	Small ASD (6 mm L to R, poor view), No visible VSD
Shunts	No PDA
Aortic Arch	Left arch, No COA
Recommendations	TEE for better ASD evaluation, Thoracic MRI for chest space lesion, Mild PE, No PLE
**CT Angiography**	
Cardiac Position/Orientation	Viscerocardiac situs solitus, D-loop, AV and VA concordance, normally related great vessels
Cardiac Structures	No ASD, No VSD, No PDA
Aortic Arch	Left-sided, No COA
Coronary Arteries	Normal origin and course
Pulmonary Arteries	MPA: 23 mm, RPA ostium: 17 mm, LPA ostium: 15 mm, No PPS
Pulmonary Veins	All PVs connect to LA
Systemic Veins	SVC and IVC connect to RA, No LSVC
Thymus	Present
Elongation & Interposition	Left pulmonary parenchyma interposition between AP window, indicative of partial absence of pericardium
**MRI Imaging**	
Cardiac Morphology	LA: 14 cm², RA: 12.5 cm²
Cardiac Function	LV: LVEF = 61%, LVEDV = 76.6 mL/m², LVMi = 39 g/m², ESVI = 29 mL/m², Stroke volume: 97 mL, CO: 5.4 L/min, CI: 2.7 L/min/m², RV: RVEF = 58%, RVEDVi = 84 mL/m², RVESVi = 35 mL/m², Stroke volume: 49 mL, CO: 5.6 L/min, CI: 2.6 L/min/m²
Velocity Flow Mapping	Aortic and Pulmonic valves: Normal excursion and coaptation, No significant stenosis or regurgitation, Mild MR, Trivial TR
Gadolinium Study	No myocardial inflammation or significant pericardial effusion, Signal intensity ratio: 1.3–1.7 (Normal < 1.9), No pathologic enhancement
Elongation & Interposition	Evidence of leftward displacement and elongation of the ventricular chambers, suggestive of left-sided pericardial agenesis. MRI confirms the absence of the pericardium without evidence of myocardial abnormalities, pericardial effusion, or herniation.

**Abbreviations**: AO (Aorta), AP (Anteroposterior), ASD (Atrial Septal Defect), AV (Atrioventricular), CI (Cardiac Index), CO (Cardiac Output), COA (Coarctation of the Aorta), CT (Computed Tomography), CXR (Chest X-ray), D-loop (Dextrorotation loop), EF (Ejection Fraction), ESVI (End-Systolic Volume Index), G (Gradient), IVC (Inferior Vena Cava), LA (Left Atrium), LPA (Left Pulmonary Artery), LSVC (Left Superior Vena Cava), LV (Left Ventricle), LVEDV (Left Ventricular End-Diastolic Volume), LVEF (Left Ventricular Ejection Fraction), LVH (Left Ventricular Hypertrophy), LVMi (Left Ventricular Mass Index), MPA (Main Pulmonary Artery), MPG (Mean Pressure Gradient), MRI (Magnetic Resonance Imaging), MR (Mitral Regurgitation), NRGAS (Normal Right Atrial and Great Arteries), PA (Pulmonary Artery), PDA (Patent Ductus Arteriosus), PE (Pericardial Effusion), PI (Pulmonary Insufficiency), PS (Pulmonary Stenosis), PPg (Peak Pressure Gradient), PV (Pulmonary Vein), Qp/Qs (Pulmonary to Systemic Flow Ratio), RA (Right Atrium), RAE (Right Atrial Enlargement), RV (Right Ventricle), RVEDVi (Right Ventricular End-Diastolic Volume Index), RVEF (Right Ventricular Ejection Fraction), RVESVi (Right Ventricular End-Systolic Volume Index), RVH (Right Ventricular Hypertrophy), RPA (Right Pulmonary Artery), STIR (Short Tau Inversion Recovery), SVC (Superior Vena Cava), TAPSE (Tricuspid Annular Plane Systolic Excursion), TAV (Tricuspid Aortic Valve), TEE (Transesophageal Echocardiogram), TR (Tricuspid Regurgitation), VSD (Ventricular Septal Defect), VTI (Velocity-Time Integral)

**Fig. 5:**
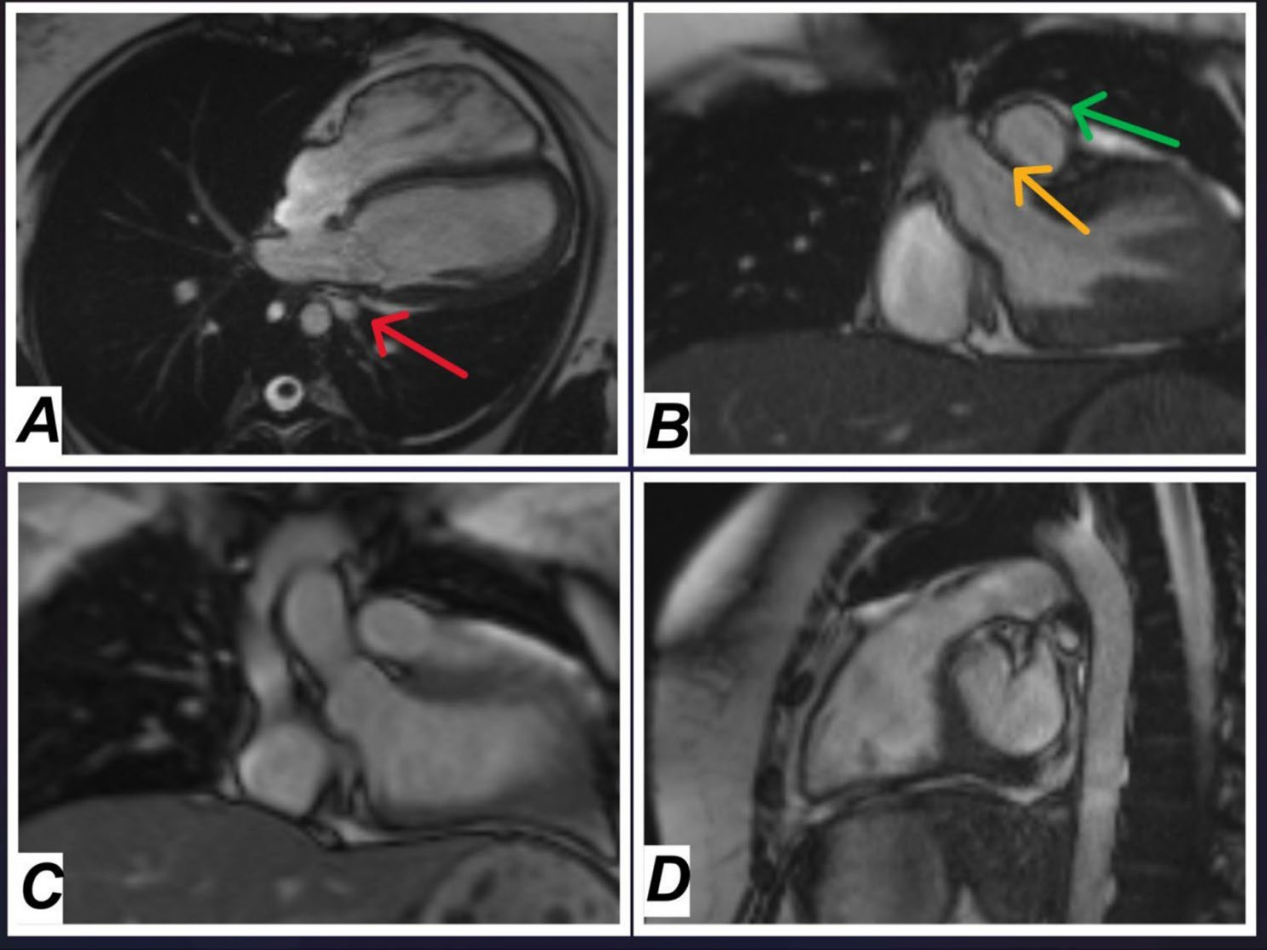
Cardiac MRI confirmed the diagnosis of unilateral agenesis **A**: Four-chamber view showing narrowing of the caliber of the left inferior pulmonary vein (red arrow) due to compression between the descending aorta and the left atrium, caused by excessive levorotation of the heart secondary to left-sided pericardial agenesis. **B** and **C**: Coronal views showing the absence of the left pericardium, along with hyperinflation of the right lung and reduced volume of the left lung. The exaggerated shift of the heart towards the left is evident. The right arrow shows the part of the heart with a pericardium; the yellow one specifies the side where the pericardium terminates. **D**: Sagittal view demonstrates the left pericardium’s absence, with the heart shifting toward the left hemithorax due to the missing pericardium

Finally, the definitive imaging modality was cardiac magnetic resonance imaging (MR). The MRI confirmed the diagnosis of complete left-sided pericardial agenesis. It provided detailed images showing the absence of the pericardium, leftward displacement of the heart, and narrowing of the left inferior pulmonary vein due to compression between the descending aorta and the left atrium, as seen in the four-chamber view (Fig. 5: A). The short-axis and coronal views (Fig. 5: B, C, and D) further demonstrated the absence of the pericardium, the exaggerated leftward shift of the heart, and the hyperinflation of the right lung. Additionally, the MRI ruled out any myocardial abnormalities, pericardial effusion, or herniation, which are potential complications of pericardial agenesis (Fig. 5: A-D) and (Table 1).

## Conclusion and follow-up

After a comprehensive evaluation, including imaging and laboratory tests, the patient was diagnosed with complete left-sided pericardial agenesis, a rare congenital condition. As the patient was asymptomatic aside from the initial chest pain, and no complications such as pericardial effusion, herniation, or coronary artery compression were detected, a conservative management approach was chosen. The patient was reassured about the generally benign nature of her condition and was discharged with clear instructions to monitor for any warning signs, such as increasing chest pain, shortness of breath, palpitations, syncope, or any new or worsening symptoms. She was advised to avoid strenuous physical activity until further evaluation.

For ongoing care, a follow-up visit was scheduled with her cardiologist in three months to monitor her condition, with an additional echocardiogram planned in six months to reassess her heart’s position and function. The patient and her family were educated on recognizing symptoms that could indicate complications and were instructed on when to seek immediate medical attention.

## Discussion

Symptomatic congenital pericardial agenesis is a rare clinical entity. Its symptoms are often nonspecific and overlap with other common conditions, making diagnosis challenging​ [7]. In our case, the patient presented with isolated chest pain, a common but nonspecific symptom, highlighting the subtlety with which this condition can manifest.

Congenital pericardial agenesis arises from a failure of the pleuropericardial membranes to fuse completely during the fifth week of gestation, resulting in the partial or complete absence of the pericardium​ [5]. This absence compromises the pericardium’s protective and supportive functions, increasing the risk of cardiac positional shifts within the thoracic cavity. Such displacement can place mechanical strain on the heart and great vessels, potentially leading to chest pain, especially during physical activity or changes in body position [8].

Patients with this condition often present with nonspecific symptoms such as chest pain, dyspnea, or palpitations, making diagnosis challenging. Imaging studies, including chest X-rays and CT scans, can reveal characteristic signs that help confirm the diagnosis of congenital pericardial agenesis [4, 9]. One such diagnostic clue is the ‘Snoopy sign,’ which indicates the complete absence of the left pericardium and is identified by the distinctive appearance of the cardiac silhouette on imaging [10]. According to the 2015 ESC Guidelines for diagnosing and managing pericardial diseases, multimodality imaging, including cardiac magnetic resonance (CMR) and echocardiography, is essential for a comprehensive evaluation and accurate diagnosis of pericardial conditions. These modalities confirm the diagnosis and assess the extent of the defect and its impact on cardiac function, guiding appropriate management strategies [11].

Management typically depends on the presence of symptoms; asymptomatic isolated agenesis usually requires no treatment. However, symptomatic patients, particularly those with the complete form of agenesis, may benefit from surgical intervention such as pericardioplasty to alleviate symptoms [1]. In cases of partial pericardial absence, surgery may be indicated to prevent complications such as herniation, type A aortic dissection, or coronary artery compression [6]. Partial pericardial defects can lead to serious complications, including cardiac herniation, which may result in critical conditions such as left atrial appendage strangulation or coronary artery compression, often requiring surgical intervention [5, 12]. Moreover, pericardial agenesis can precipitate significant complications like pericardial effusion or cardiac tamponade, both of which are potentially life-threatening and demand immediate diagnosis and management [11]. The prognosis of pericardial agenesis largely depends on the extent of the defect;

In contrast, complete agenesis typically has a benign course with a favorable outcome; partial agenesis carries a heightened risk of complications, necessitating closer monitoring and potential surgical intervention​ [1, 12]. Additionally, right ventricular enlargement and associated cardiac abnormalities in partial agenesis complicate the clinical course, underscoring the need for early and accurate diagnosis. The absence of the pericardium can cause excessive heart motion, leading to mechanical stress and increasing the risk of arrhythmias and other functional issues [13].

A study by Khayata et al. involving eight patients with congenital pericardial defects found that partial defects were more common on the left side and that right ventricular dilation was a frequent echocardiographic finding. The study also highlighted that most patients remained stable and asymptomatic during follow-up, with only one requiring surgical intervention. These findings underscore the variability in clinical outcomes, further emphasizing the need for individualized management strategies based on the extent of the pericardial defect and associated symptoms [14].

In a case report by Trimarchi et al., a 32-year-old man with complete left-sided pericardial agenesis was asymptomatic and had a good prognosis. The study emphasized that in asymptomatic cases of complete pericardial agenesis, no treatment is typically required, and a comprehensive diagnostic work-up with multimodality imaging plays a crucial role in preventing diagnostic errors and optimizing patient follow-up. However, the study also noted that partial pericardial defects are associated with a higher risk of complications, such as herniation and arrhythmias. It may require closer monitoring and, in some cases, surgical intervention [6].

In a case study by Kalekar et al., a 32-year-old male presented with chest discomfort and radiating pain that mimicked myocardial infarction. Multimodal imaging, including chest X-ray, echocardiography, and cardiac MRI, revealed complete left pericardial agenesis, characterized by significant leftward displacement of the heart. This case underscores the importance of considering pericardial agenesis in the differential diagnosis of chest pain, particularly when initial investigations are inconclusive, and highlights the critical role of advanced imaging techniques in diagnosing this rare condition [15].

The evidence summarized in the literature review table supports this approach. It compares clinical presentations, diagnostic methods, and management strategies across different studies (Table 2).

**Table 2:** Summary of Case reports on Pericardial Agenesis

Author (YOP)	Gender & Age	CC, HX, PH/E, Imaging	Dx, Tx, Prog
Trimarchi G. (2024)[6]	M, 32	CC: Asymptomatic, found through occupational health visit due to ECG abnormalities. HX: No significant medical history, except for class 2 obesity. PH/E: Systolic ejection murmur, no signs of oedema or congestion. Imaging: Altered LV shape on TTE, leftward shift of heart on X-ray, confirmed pericardial agenesis via MRI.	Dx: Complete left-sided pericardial agenesis. Tx: Loop-recorder implantation, 6-month follow-up, no immediate treatment. Prog: Good, no surgical intervention needed.
D’Arma GMA. (2024)[16]	F, 24	CC: Asymptomatic, incidental finding. HX: No significant medical history or family history of sudden cardiac death. PH/E: Normal, with bradycardia. Imaging: CMR showed complete dislocation of heart with no evidence of pericardium; ECG showed right axis deviation.	Dx: Complete pericardial agenesis. Tx: No treatment needed; regular follow-up recommended. Prog: Good, patient remains asymptomatic.
Pimienta-Ibarra A. (2024)[17]	F, 23	CC: 4-month hx of progressive dyspnea, cough with purulent sputum. HX: Initially treated for community-acquired pneumonia with no improvement. PH/E: None significant. Imaging: CT showed a cystic mass in the left upper lobe, suspected bronchogenic cyst; Intraoperative finding of absent pericardial sac.	Dx: Bronchogenic cyst with complete left pericardial agenesis. Tx: Upper lobectomy via VATS, post-op management with ABx. Prog: Good, conservative management for agenesis.
Kalekar T. (2023)[15]	M, 32	CC: Chest discomfort, radiating pain to back and left shoulder, worsened after weight training. HX: Recurrent similar complaints since age 10. PH/E: Normal, except for tachycardia and tachypnoea. Imaging: CXR shows left lateral position of heart; MRI confirms left pericardial agenesis with ASD.	Dx: Left pericardial agenesis with secundum-type ASD. Tx: Conservative management with regular follow-up, advised against heavy lifting. Prog: Good, no immediate intervention needed.
Alyami B. (2022)[18]	M, 40	CC: Recurrent non-radiating substernal chest pain. HX: History of hyperlipidemia and SVT. PH/E: Normal, except for sinus tachycardia, RBBB on ECG. Imaging: CXR shows leftward shift of heart, CTA confirms CAP with levoposition of heart.	Dx: Complete congenital absence of pericardium (CAP). Tx: Conservative, no intervention needed. Prog: Asymptomatic, regular follow-up advised.
Bernardinello V. (2020)[13]	M, 28	CC: Asymptomatic, ECG abnormalities found during preparticipation screening. HX: Competitive runner with no significant medical history. PH/E: Normal. Imaging: Echo showed RV enlargement; CMR confirmed leftward dislocation of the heart; CT confirmed incomplete pericardial agenesis.	Dx: Incomplete pericardial agenesis. Tx: No treatment needed; regular follow-up recommended. Prog: Good, patient remains asymptomatic.
Oliveira CC. (2020)[19]	M, 58	CC: Persistent atypical chest pain. HX: No significant medical history. PH/E: Sinus bradycardia, right axis deviation, incomplete RBBB. Imaging: Echo shows teardrop-shaped heart; CMR shows leftward rotation of the heart and interposition of lung tissue.	Dx: Complete pericardial agenesis. Tx: Conservative management, no intervention required. Prog: Asymptomatic, regular follow-up advised.

**Abbreviation**: ABx: Antibiotics, ASD: Atrial Septal Defect, CAP: Congenital Absence of Pericardium, CC: Chief Complaint, CMR: Cardiac Magnetic Resonance, CTA: Computed Tomography Angiography, CXR: Chest X-ray, Dx: Diagnosis, Echo: Echocardiogram, ECG: Electrocardiogram, Hx: History, LV: Left Ventricle, MRI: Magnetic Resonance Imaging, PH/E: Physical Examination, Prog: Prognosis, RBBB: Right Bundle Branch Block, RV: Right Ventricle, SVT: Supraventricular Tachycardia, Tx: Treatment, TTE: Transthoracic Echocardiogram, VATS: Video-Assisted Thoracoscopic Surgery, YOP: Year of Publication

## Conclusion

Though rare, congenital pericardial agenesis presents diagnostic challenges due to its often asymptomatic nature and nonspecific symptoms. Clinicians should be vigilant in cases of unexplained chest pain, especially when standard tests are inconclusive. Advanced imaging, such as cardiac MRI and echocardiography, is essential for diagnosis and management. While complete agenesis usually has a benign course, partial defects carry a higher risk of complications, necessitating close monitoring and potential surgical intervention. Early recognition and individualized management are key to preventing serious complications in patients with atypical cardiac presentations.

## Data Availability

No datasets were generated or analysed during the current study.
